# A Novel Flavi-like Virus in Alfalfa (*Medicago sativa* L.) Crops along the Snake River Valley

**DOI:** 10.3390/v14061320

**Published:** 2022-06-16

**Authors:** Jennifer Dahan, Yuri I. Wolf, Gardenia E. Orellana, Erik J. Wenninger, Eugene V. Koonin, Alexander V. Karasev

**Affiliations:** 1 Department of Entomology, Plant Pathology and Nematology, University of Idaho, Moscow, ID 83844, USA; jdahan@uidaho.edu (J.D.); gardeniao@uidaho.edu (G.E.O.); 2 National Center for Biotechnology Information, National Library of Medicine, National Institutes of Health, Bethesda, MD 20894, USA; wolf@ncbi.nlm.nih.gov (Y.I.W.); koonin@ncbi.nlm.nih.gov (E.V.K.); 3 Department of Entomology, Plant Pathology and Nematology, University of Idaho, Kimberly Research and Extension Center, Kimberly, ID 83341, USA; erikw@uidaho.edu

**Keywords:** virus emergence, virus evolution, virus detection

## Abstract

Alfalfa is an important perennial forage crop in Idaho supporting dairy and cattle industries that is typically grown in the same field for as many as 4 years. Alfalfa stands of different ages were subjected to screening for viruses using high-throughput sequencing and RT-PCR. The two most common viruses found were alfalfa mosaic virus and bean leafroll virus, along with *Medicago sativa* amalgavirus, two alphapartitiviruses, and one deltapartitivirus. Additionally, a new flavi-like virus with an unusual genome organization was discovered, dubbed Snake River alfalfa virus (SRAV). The 11,745 nt, positive-sense (+) RNA genome of SRAV encodes a single 3835 aa polyprotein with only two identifiable conserved domains, an RNA-dependent RNA polymerase (RdRP) and a predicted serine protease. Notably, unlike all +RNA virus genomes in the similar size range, the SRAV polyprotein contained no predicted helicase domain. In the RdRP phylogeny, SRAV was placed inside the flavi-like lineage as a sister clade to a branch consisting of hepaci-, and pegiviruses. To the best of our knowledge, SRAV is the first flavi-like virus identified in a plant host. Although commonly detected in alfalfa crops in southern Idaho, SRAV sequences were also amplified from thrips feeding in alfalfa stands in the area, suggesting a possible role of *Frankliniella occidentalis* in virus transmission.

## 1. Introduction

Alfalfa (*Medicago sativa* L.) is an important forage crop grown on more than 400,000 ha in the State of Idaho in support of the burgeoning cattle and dairy industries. In terms of acreage, it is the second most widely grown of all Idaho crops, behind wheat [1]. In Idaho, alfalfa is grown in the same field for multiple seasons, typically about four, but may stay in the same field even longer. Insecticides are rarely applied to forage alfalfa fields in Idaho. Alfalfa forage production is characterized by up to four cuttings per summer, with subsequent use for hay and haylage. Hence, alfalfa represents a perfect sentinel crop accumulating pathogens, including viruses, over multiple growing seasons, with plenty of opportunities for mechanical transmission of viruses during the repeating cycles of hay cutting, which also facilitate insect dispersal and vector transmission. These agricultural practices for alfalfa production are not unique to Idaho and are similar to those implemented all over the world.

Prior to the introduction of HTS methodologies, the six most common viruses were reported to affect alfalfa production worldwide, with another 14 occasionally found to infect alfalfa but not having a significant impact on the crop [2]. The four most common viruses found in alfalfa in North America are alfalfa mosaic virus (AMV, genus *Alfamovirus*), bean leafroll virus (BLRV, genus *Luteovirus*), pea streak virus (PeSV, genus *Carlavirus*), and red clover vein mosaic virus (RCVMV, genus *Carlavirus*), all of them single-stranded (ss), positive-sense (+) RNA viruses that are aphid-transmitted [2]. Two other common viruses found outside of North America are alfalfa enation virus (AEV, genus *Nucleorhabdovirus*) and lucerne transient streak virus (LTSV, genus *Sobemovirus*), which are transmitted by aphids and beetles, respectively [2]; the latter has been recently described in the U.S in thrips and alfalfa [3,4]. With the advent of HTS, systematic study of the alfalfa virome became possible and yielded multiple known and new plant viruses identified in different countries over the last several years [3,4,5,6,7,8,9,10,11,12,13,14]. In addition to the common viruses known to infect alfalfa and other crops, a group of persistent viruses was identified in alfalfa, including Medicago sativa alphapartitiviruses 1 and 2 (MsAPV1 and MsAPV2), Medicago sativa deltapartitivirus 1 (MsDPV1), and Medicago sativa amalgavirus 1 (MsAV1) [5,6,13,14,15,16,17,18]. Although several viruses, such as AMV, BLRV, and persistent viruses, are common in alfalfa crops grown around the world, many viruses identified in alfalfa using HTS display a more limited, localized distribution characteristic of a specific country or even a smaller geographic location [5,6,7,8,9,10,11,12,13]. Given the perennial nature of alfalfa stands, these occasional finds of various plant viruses normally associated with other crops seem to suggest a role of alfalfa as a reservoir of pathogens, potentially threatening agricultural production of many crops [4,5]. Additionally, because most alfalfa-infecting viruses have insect vectors, HTS was also applied for virome studies of insect pests found in alfalfa fields, such as thrips, aphids [18], and weevils [8], and in addition to plant viruses ingested by the insects [18], multiple sequences from arthropod viruses were detected [8]. The majority of the reads assigned to arthropod viruses in alfalfa weevils belong to the families *Iflaviridae* and *Solemoviridae* [8].

In the United States, studies of the alfalfa virome using HTS have led to the identification of new viruses, such as alfalfa virus S (AVS, genus *Allexivirus*) in the State of Washington [19], and have contributed to a more accurate description of the complex viral populations in alfalfa [4,19]. Here, we describe virome analysis of alfalfa samples from southern Idaho collected in an irrigated 4-year-old alfalfa field. These samples yielded several ssRNA and double-stranded (ds) RNA viruses common in alfalfa and found on different continents. In addition to this previously characterized, typical repertoire of viruses, a novel virus was discovered through analysis of the HTS data, with a complete genome of 11,745 nt, provisionally named Snake River alfalfa virus (SRAV). Comparative genomic and phylogenetic analyses show that SRAV is the first flavi-like virus infecting a plant host to be identified. This virus was found to be common in the sampled alfalfa fields in south-central Idaho.

## 2. Materials and Methods

### 2.1. Alfalfa Sampling and Sample Processing

Two locations in south-central Idaho were sampled—one in Minidoka County and another in Twin Falls County—approximately 80 km apart on opposite banks of the Snake River. Location 1 near Rupert (ID) in Minidoka County represented a single standard, 50-ha irrigated production field with an alfalfa stand planted in Spring 2017. It was in its fourth growing season and was sampled in July 2020. Location 2 in Twin Falls County represented experimental grounds of the Kimberly Research and Extension Center, University of Idaho, and included five relatively small, 0.4–2.2 ha fields planted with alfalfa between 2013 and 2020; this location was sampled twice: in July 2021 and again in August 2021.

Samples were collected randomly in the field from alfalfa plants visually assessed to exhibit mosaic, yellowing, vein clearing, leaf distortions, or growth retardation. A sample represented one complete alfalfa stem with all leaves intact but without roots; this entire stem was placed in a plastic bag and kept in a cooler on ice for transport to the laboratory. The typical time between alfalfa leaf tissue collection and nucleic acid extraction was between 3 and 7 days. Tissue aliquots for each sample were stored at −80 °C until further use.

In August 2021, concomitantly with the collection of alfalfa samples, 20 thrips (*Frankinella occidentalis*) were collected from two alfalfa fields, alongside three pea aphids (*Acyrthosiphon pisum*). A summary of all the samples collected and subjected to analysis is presented in Table 1. All these samples were shipped to the University of Idaho laboratory (Moscow, ID, USA) by an overnight mail service and analyzed immediately.

### 2.2. Mechanical Inoculations of Nicotiana benthamiana and Common Bean Plants

Alfalfa samples collected in August 2021 were tested for the presence of alfalfa viruses using RT-PCR (see below) and were also used as a source of inoculum of laboratory plants in the greenhouse. Each of the ten collected alfalfa samples was mechanically inoculated into three *N. benthamiana* and two *Phaseolus vulgaris* cv. Dubbele Witte plants using standard protocols [20,21]. Inoculated *N. benthamiana* and common bean plants were observed for eight weeks post-inoculation and tested for the alfalfa viruses using RT-PCR at 5 (*N. benthamiana*, bean) and 8 weeks (*N. benthamiana*) post-inoculation (wpi) (upper, non-inoculated leaves).

### 2.3. RNA Extraction and HTS Analysis

About 200 mg of each of the alfalfa samples collected in July 2020 was ground using a mortar and pestle in liquid nitrogen, and total RNA was extracted with a Spectrum Plant total RNA kit (Sigma-Aldrich, St. Louis, MI, USA). Resulting RNAs were subjected to DNase treatment using a DNAse Max kit (Qiagen, Hilden, Germany) and subsequently concentrated using an RNA clean and concentration kit—5 (ZymoResearch, Irvine, CA, USA). Ribosomal RNAs were then depleted using a RiboMinus plant ribodepletion kit (ThermoFisher Scientific, Waltham, MA, USA), and resulting ribodepleted RNAs were concentrated by ethanol precipitation. Between 340 and 420 ng of ribodepleted RNA per sample was then subjected to strand-specific library preparation using a Kapa RNA HyperPrep kit (Roche, Basel, Switzerland) and sequencing in paired-end 300 bp read format on a MiSeq platform (Genomics and Bioinformatics Resources Core, IIDS, University of Idaho).

Resulting paired-end reads were cleaned using Trimmomatic v0.38 in ILLUMINACLIP mode with the following settings: 2:30:10:8:TRUE LEADING:3 TRAILING:3 SLIDINGWINDOW:4:20 MINLEN:100 [22]. Paired, trimmed reads were assembled using SPAdes v3.14.0 in rnaspades mode [23]. Resulting contigs were then submitted to a BLASTn search against the nt_v5 database (as of 6 August 2020), with a cut-off e value of 0.00001 and a max_target_seqs setting of 1. Contigs with no hits were then submitted to BLASTx, and all contigs with viral hits were then extracted and analyzed further in Geneious Prime v2021.1.1 (Biomatters, Ltd., Auckland, New Zealand).

### 2.4. Nucleic Acid Extraction, RT-PCR Testing, and Sanger Sequencing

For alfalfa and insect samples collected in July and August 2021, total virus nucleic acids were extracted from alfalfa according to the protocol described by Dellaporta et al. [24]. Reverse transcription was performed using 4.5 µL of the total nucleic acid extract in a 25 µL reaction mixture that contained 5× first-strand buffer (Promega, Madison, WI, USA), 2.5 mM dNTP, 3 µM Oligo dT + random hexamers, rRNasin ribonuclease inhibitor (Promega), and M-MLV reverse transcriptase (Promega). Before the reverse transcription reaction, the RNA template was incubated at 70 °C for 5 min; then, the reverse transcription mix was added. The profile used consisted of incubation at 37 °C for 60 min and reverse transcriptase deactivation at 70 °C for 10 min. All PCR reactions were accomplished by GreenTaq (GenScript, Piscataway, NJ, USA) in a 20 µL reaction mixture that contained 10× GreenTaq buffer, 2.5 mM dNTP, 5 µM of each forward primer and reverse primer, GreenTaq, and 2 µL cDNA template. The PCR profile consisted of denaturing at 94 °C for 2 min and 35 cycles of 94 °C for 30 s, 55–65 °C for 30 s (depending on the melting temperature of primers used), and 72 °C for 1 to 2 min (depending on the fragment length amplified), followed by a final extension for 10 min at 72 °C. Sanger sequencing was performed on a series of overlapping RT-PCR fragments [25] amplified on total RNA extracted from the infected alfalfa plants as described above. Primers used to amplify these DNA fragments are listed in Supplemental Table S1. PCR fragments were treated with ExosapIt (Affymetrix, Cleveland, OH, USA) and submitted for sequencing to Elim Biopharmaceuticals, Inc. (Hayward, CA, USA). The 5′ terminus was amplified using a 5′/3′ RACE kit (Roche, Indianapolis, IN, USA) according to the manufacturer’s instructions using primers SP1, SP2, and SP3 (Supplementary Table S1); the amplified PCR product was cloned into *E. coli* using a pGEM-T Easy vector system (Promega) and sequenced using M13 primers. The 3′ terminus was amplified using SP5 primer (Supplementary Table S1), then cloned and sequenced with M13 primers. The obtained contigs were assembled using Geneious Prime v2021.1.1 (Biomatters).

### 2.5. Sequence and Phylogenetic Analysis

For analysis of the newly discovered flavi-like virus, protein sequence database searches were performed using PSI-BLAST and HHPred [26]. Sequences of the RNA-dependent RNA polymerases (RdRPs) of flavi-like viruses were extracted from GenBank as of May 2020 and were used to construct a phylogenetic tree and assess the taxonomic position of the new putative alfalfa virus. The protein sequences were aligned using MUSCLE5 [27]; positions containing more than a 0.667 fraction of gap characters and homogeneity below 0.05 [28] were trimmed from the alignment. Phylogeny was inferred using IQ-tree 2 software v2.1.3 [29] with the Q.pfam+F+R8 model, as selected by ModelFinder [30], and aBayes branch support [31]. The tree was rooted at the Phytophthora infestans RNA virus 1, which belongs to the sister clade of *Flaviviridae* (the clade denoted as f.0072 in [32]).

## 3. Results

**Figure 1:**
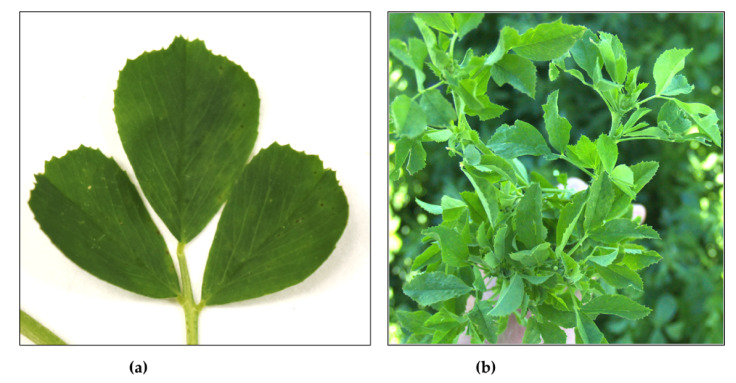
Mosaic, vein clearing, and flecking on the foliage of alfalfa samples ALF1060 (**a**) and ALF1061 (**b**) collected from location 1 in July 2020.

Visual assessments of alfalfa stands during the summers of 2020 and 2021 were conducted randomly. Only plants exhibiting visible foliar symptoms of mosaic, vein clearing, flecking, leaf distortions, and overall growth retardation were sampled for further laboratory studies. Examples of the symptoms observed are presented in Figure 1. Plant ALF1060 (Figure 1a) was eventually found positive for AMV and SRAV, whereas plant ALF1061 (Figure 1b) was positive for AMV, BLRV, and SRAV. Six samples were collected from location 1 in July 2020; five samples from each of the five fields were collected from location 2 in July 2021, for a total of 25 samples, and an additional five samples from each of the two fields were collected from location 2 in August 2021, for a total of 10 samples.

### 3.1. Viruses Identified in the 2020 Alfalfa Crop by High-Throughput Sequencing

Five of the six samples collected from location 1 in July 2020 were subjected to HTS analysis on the MiSeq platform. Between 6702 and 7560 contigs over 1 kb per sample were assembled from each of the five samples (Supplemental Table S2) and analyzed using BLASTn and BLASTx programs. Sequences specific to six viruses known to infect alfalfa were identified in these five samples based on HTS data (Table 2). These were AMV (three genome components), BLRV, MsAPV1 (two genome components), MsAPV2 (two genome components), MsDPV1 (two genome components), and MsAV1. The abundance of the virus-specific reads differed between the five samples, indicating that AMV was, by far, the most abundant virus present in all five samples (Table 2). BLRV was identified in two of the five samples by HTS, MsAPV2 in one, MsDPV1 in three, and MsAV1 in four (Table 2). Based on the sizes of the contigs recovered from the five alfalfa samples, it appeared that nearly complete genomes or individual genome components were assembled for all six common alfalfa viruses; their sequences were deposited in the GenBank database under the accession numbers listed in Supplementary Table S3. The overall nucleotide sequence identities for each of the viruses between individual samples were found at 99–100% and between virus sequences assembled from the five alfalfa samples and the closest matches from the GenBank database, within the same 99–100% range for the vast majority of the identified virus contigs (Supplementary Table S3).

However, there were three exceptions. Among the various virus-related sequences, an 11,803 nt contig was identified in sample ALF1060 (see Figure 1a), and a 11,797 nt contig was detected in sample ALF1071 (Table 2). Both contigs encompassed a single, 11,503 nt-long open reading frame (ORF), which encoded a 3835 aa polyprotein. These two large contigs from ALF1060 and ALF1071 were 99.9% identical at the nucleotide sequence level, with only 14 SNPs between the two. A 7788 nt contig was assembled from sample ALF1061 representing the 3′-terminal portion of the same sequence found in ALF1060 and ALF1071 (Table 2). None of these three contigs contained a poly(A) tail.

A BLASTn search of the GenBank database returned no similar nucleotide sequences. A BLASTp search of the +RNA virus subset of the NCBI non-redundant protein sequence database (as of 20 April 2022) yielded a significant alignment (expectation value 2 × 10^−4^; 21% identity over an amino acid sequence alignment containing 265 positions with a 450 amino acid C-terminal region of the polyprotein of Bole tick virus 4 (GenBank Accession number QUJ17980 [33])) containing the four amino acid motifs (including the GDD signature of the catalytic site) diagnostic of the +RNA virus RdRPs (Supplementary Figure S1). Subsequent PSI-BLAST iterations demonstrated significant similarity with the RdRP-containing regions of the polyproteins of many flavi-like viruses that have been recently identified by metagenomic approaches in arthropod holobionts [28]. A more sensitive HHPred search, which employs a profile-against-profile search to detect distant protein sequence similarities, detected significant similarity to several RdRP families, the top hit being bovine virus diarrhea pestivirus RdRP (probability: 98%).

In addition to the RdRP, the HHPred search detected an approximately 100 amino acid region in the middle of the polyprotein with moderate similarity to the serine proteases of arteriviruses and flaviviruses (probability of 73% and 63%, respectively). Despite the limited sequence conservation, this region of the new virus polyprotein contained conserved histidine and serine residues corresponding to the catalytic residues of the virus proteases, with the catalytic serine embedded with a characteristic GxSGG motif. The secondary structure prediction for this portion of the polyprotein was also fully compatible with the known protease structures (Supplementary Figure S2), indicating that the virus polyprotein contains an active serine protease domain. Given the presence of the RdRP and protease domains in the encoded polyprotein and the size of the contig compatible with the typical genome size of flavi-like viruses, we hypothesized that this 11,803 nt contig represented a complete or nearly complete genome of a novel +RNA virus provisionally named “Snake River alfalfa virus” (SRAV). The name is based on the geographic area where this virus was originally found, i.e., along the Snake River Valley, ID, USA.

To confirm and validate the presence of the SRAV sequences in the original samples collected in July 2020, RT-PCR assays were conducted on the six original alfalfa samples using SRAV-specific primers (Supplementary Table S1) designed based on the HTS-derived sequence. Four out of the six tested 2020 alfalfa samples were found to be SRAV-positive by RT-PCR, and the three amplified 609/713/843 nt fragments had 99.9–100% identity to the HTS-derived sequence when subjected to conventional Sanger sequencing (Table 3). The presence of AMV was confirmed in all six of the tested 2020 alfalfa samples, matching the HTS data, whereas BLRV was confirmed in four of the six samples (Table 3). Interestingly, BLRV was unambiguously identified by RT-PCR in samples ALF1059 and ALF 1061 (Table 3), whereas HTS identified only a few BLRV-specific reads (Table 2). This may indicate that RT-PCR had a better sensitivity in terms of virus detection in alfalfa than the HTS strategy. The persistent alfalfa viruses with dsRNA genomes, MsAPV1, MsAPV2, MsDPV1, and MsAV1, were also easily identified by RT-PCR in all positive samples based on the HTS data (Table 2 and Table 3). Partial sequences determined for the persistent viruses using conventional Sanger sequencing were found to be 100% identical to the HTS-derived sequences.

**Figure 2:**
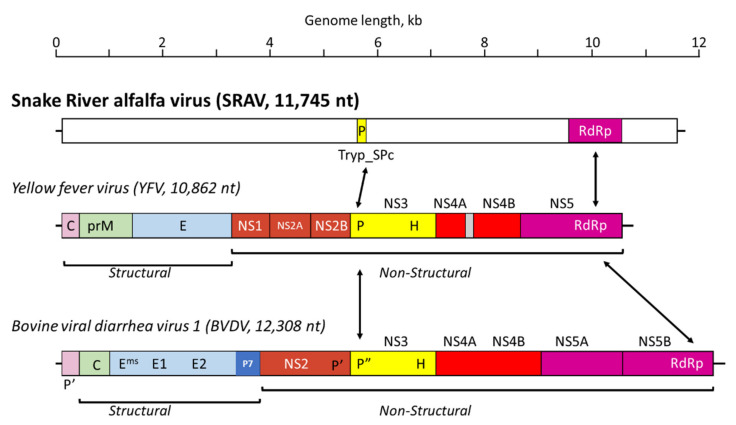
Schematic representation of the Snake River alfalfa virus (SRAV) genome in comparison to the genome organization of yellow fever virus (YFV, flavivirus) and bovine viral diarrhea virus (BVDV, pestivirus). Homologous protein domains in all three polyproteins, Tryp_SPc (trypsin-like serine protease) and RdRP (RNA-dependent RNA polymerase), are indicated by arrows.

We then undertook a more thorough molecular and bioinformatics analysis of the SRAV genome and polyprotein. The conceptual translation of the complete SRAV genome produced a single, large open reading frame encoding a 3835 aa polyprotein (Figure 2).

**Figure 3:**
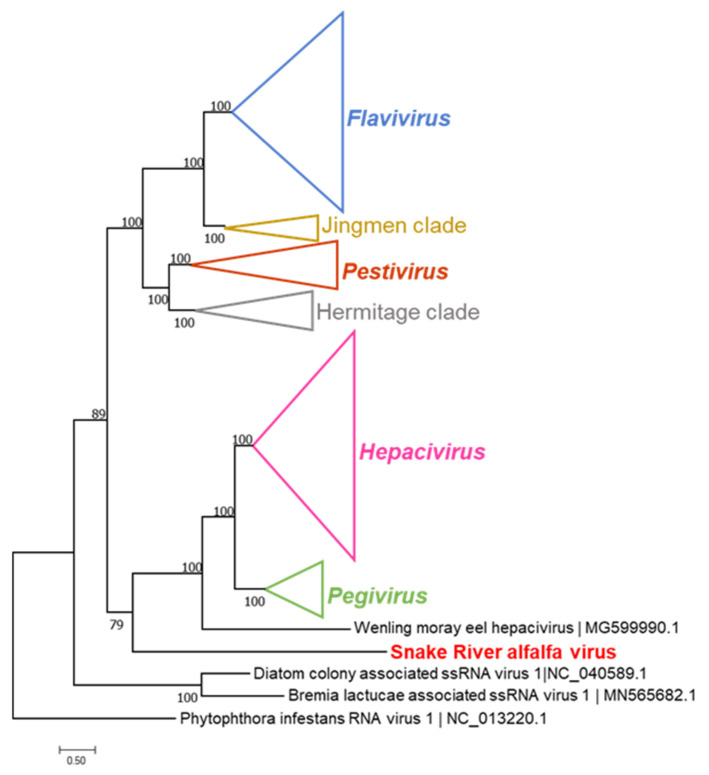
Phylogenetic analysis of the RdRP domains of flavi-like viruses and phylogenetic placement of the newly described Snake River alfalfa virus (SRAV). Numbered nodes indicate aBayes support values; large clades are collapsed and depicted as triangles. Virus genera of *Flaviviridae* currently approved by the International Committee on Taxonomy of Viruses (ICTV) are shown in bold italic; two clades of unclassified arthropod flavi-like viruses are provisionally denoted as “Jingmen” and “Hermitage”. See Supplementary Figure S3 for the expanded tree.

The relative positions of the two identified conserved domains, Tryp_SPc and RdRp, within the 3835 aa polyprotein resembled the genome architecture of flavi-like viruses (see Figure 2). The genome size of SRAV, the size of the polyprotein (3385 aa), relative positions of the protease and polymerase domains in the polyprotein, and the sequence similarity in the RdRP domain all appeared consistent with the placement of SRAV among the flavi-like viruses, which was supported by phylogenetic analysis (Figure 3). Surprisingly, however, no helicase domain was found in the SRAV polyprotein, neither in the BLAST and HHpred searches nor in a targeted search for the sequence motifs that are diagnostic of the P-loop ATPase domain or specific families of helicases [34]. So far, all +RNA genomes larger than 6 kb have been found to encode a helicase, which is thought to facilitate replication of larger RNA genomes. In particular, in the flavi-like viruses, the helicase comprises the C-terminal domain of NS3, the same protein that contains the protease domain on its N terminus (Figure 2). The apparent absence of the helicase sets SRAV apart from all known +RNA viruses. Furthermore, no homologs of the capping enzyme or structural proteins of flavi-like viruses or any other +RNA viruses were detected in the SRAV polyprotein, indicating that this is an unusual, highly divergent member of the flavi-like clade. In an attempt to clarify the taxonomic position of SRAV, we searched a large data set of RNA virus genomic sequences recently identified in thousands of metatranscriptomes [32] for potential relatives of SRAVs. However, no virus proteins with significant sequence similarity were detected, leaving SRAV an orphan among the +RNA viruses.

In the phylogenetic tree of the RdRPs of flavi-like viruses (Figure 3), SRAV formed the sister clade of the branch that consisted of the members of the genera *Hepacivirus*, *Pegivirus*, and an unclassified virus from the Wenling moray eel. Together with SRAV, this branch was the sister group of another large branch that included the genera *Flavivirus* and *Pestivirus* grouped with two yet unclassified clades of ‘Jingmen’ and ‘Hermitage’ viruses (Figure 3). The deep and distinct separation of the SRAV branch from all currently established genera of flavi-like viruses (Figure 3) appears consistent with the possibility that SRAV represents a new taxon. Given the topology of the tree presented in Figure 3, it is conceivable to suggest that SRAV will eventually become the founder of a new genus or even a higher taxon within the overall hierarchy of flavi-like viruses.

### 3.2. SRAV Is Abundant in Alfalfa Stands of Different Ages in Southern Idaho

Of the 25 alfalfa samples collected in July 2021 in location 2, 15 were found to be SRAV-positive by RT-PCR (Supplementary Table S4), producing partial SRAV genome sequences that were 99–100% identical to the 2020 HTS-derived SRAV sequence. Because July 2021 samples were collected from five alfalfa fields planted between 2013 and 2020, we attempted to discern whether the number of seasons alfalfa was in the field might be related to the level of SRAV infection (Table 3). However, we observed no apparent connection between the proportion of SRAV-infected alfalfa samples taken from each field and the date of planting (Table 4). Of the 10 alfalfa samples collected in August 2021 in location 2 (from the same two fields sampled one month earlier), three were found to be SRAV-positive (Table 3), which is similar to the five SRAV-positive out of 10 samples collected from the same fields (4B and 14B) found in July (Table 3). It is worth noting that of the 41 alfalfa samples exhibiting virus-like symptoms collected in 2020 and 2021 between two collection sites located about 80 km apart from six fields planted between 2013 and 2020, 19 and 46%, respectively, were found to be positive for this novel SRAV (see Table 3), suggesting that SRAV is common and abundant in alfalfa crops in the sampled area.

Persistent viruses MsAPV1, MsAPV2, MsDPV1, and MsAV1 were also detected in alfalfa samples collected in July and August 2021 (Table 3). MsAPV1 was detected in almost all samples collected in 2020 and 2021, whereas three other dsRNA viruses were found in 20–80% of the samples (Table 3). Because all these persistent viruses are vertically transmitted and no in-season transmission occurs, in the case of MsAPV1, MsAPV2, MsDPV1, and MsAV1, this could be linked to the level of virus genome expression.

### 3.3. Sanger Resequencing of the SRAV Genomes, Acquisition of the 5′- and 3′-Termini, and Sequence Analysis

To confirm and validate the HTS-derived 11,803 nt genome sequence of SRAV in the original SRAV-positive samples, Sanger resequencing of the whole genome was performed on the SRAV-positive samples ALF1060, collected in July 2020; 7C1, collected in July 2021; and 4B4, collected in August 2021. A total of 17 SRAV-specific primer pairs were designed based on the HTS-derived sequence and used to span the SRAV genome in a series of 17 overlapping PCR fragments (Supplemental Table S1). The 5′- and 3′-terminal sequences of the SRAV genome were acquired in one SRAV-positive sample, 4B4, using the 5′- and 3′-RACE methodologies described previously. For the 5′-terminus, nine selected bacterial colonies with plasmids containing SRAV-specific, 5′-terminal inserts produced identical sequences, which resulted in the extension of the original HTS-derived sequence by 7 nt in the 5′ direction. For the 3′-terminus, four selected bacterial colonies produced identical 3′-terminal sequences. The 3′-RACE-derived sequence trimmed the original, HTS-derived 11,803 nt sequence of the SRAV genome by 65 nt. The total size of the SRAV genome in the 4B4 sample collected in August 2021 was thus found to be 11,745 nt; this complete SRAV genome was deposited in the GenBank database under the accession number ON669064. The two other corrected, nearly complete SRAV genome sequences of 11,703 nt for sample ALF1060 (collected in July 2020) and 11,698 nt for sample 7C1 (collected in July 2021) were deposited under the accession numbers ON669065 and ON669066, respectively. Thus, the complete SRAV genome contains a 118 nt 5′-untranslated region (UTR) and a 119 nt 3′-terminal UTR.

The three complete or nearly complete sequences for SRAV isolates ALF1060, 4B4, and 7C1 were compared in order to assess the genetic diversity of SRAV isolates circulating in alfalfa in south-central Idaho relative to the geography and timing of the sample collection. All three Sanger-sequenced complete or nearly complete genomes of SRAV isolates, ALF1060, 4B4, and 7C1, ranging between 11,738 and 11,745 nt in length were found to encode the single polyprotein of 3835 aa, which was identical to that translated from the HTS-derived SRAV sequence. The genomes of the three isolates were found to be 99.9% identical in their nucleotide sequence in pair-wise comparisons. It is worth noting that the three samples were collected from three different alfalfa fields separated by a distance of up to 80 km and with planting ages of between 1 and 4 years.

### 3.4. Attempts to Transmit SRAV and Detection of the Virus in Thrips Collected in Alfalfa Stands

In August 2021, concomitantly with the collection of alfalfa plant tissue, a preliminary attempt was made to address the possible mode of spread of SRAV in alfalfa. Ten alfalfa samples collected in August 2021 from location 2 were used as inocula for mechanical inoculation of *N. benthamiana* in a greenhouse, with three *N. benthamiana* plants rub-inoculated for each of the ten alfalfa samples. Three of the ten alfalfa samples were found to be SRAV-positive by RT-PCR (Table 3), and a total of nine *N. benthamiana* plants inoculated with extracts of these SRAV-positive alfalfa samples were analyzed carefully for visual symptoms and were also tested at 5 wpi and 8 wpi for systemic infection in upper non-inoculated leaves using a RT-PCR assay with SRAV-specific primers. A total of 26 of the 30 inoculated *N. benthamiana* plants developed systemic mosaic and vein clearing between 3 and 4 wpi, but none of these *N. benthamiana* plants were found to be SRAV-positive in upper non-inoculated leaves (Table 4). The experiment suggested a successful mechanical transmission of AMV from the original eight alfalfa samples carrying this virus (see Table 3). None of the 20 common bean plants inoculated with the alfalfa leaf extracts of the August 2021 samples exhibited any virus-like symptoms in the greenhouse, and none were found to be virus-positive when tested using RT-PCR at 5 wpi (data not shown).

**Figure 4:**
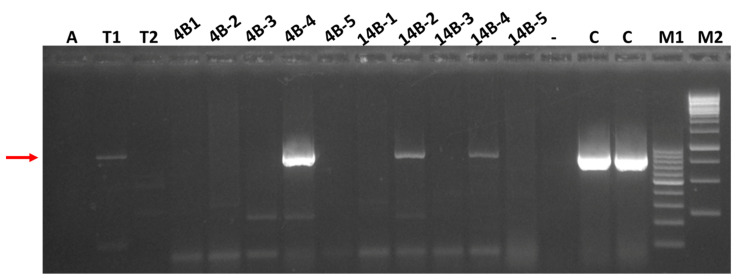
RT-PCR testing of the August 2021 alfalfa and insect samples for the presence of the Snake River alfalfa virus (SRAV); PCR products were analyzed in agarose gel and visualized under UV light. A: composite sample of the aphids collected from field 4B; T1 and T2: composite samples of thrips collected from fields 14B and 4B, respectively; the next ten samples are alfalfa samples from fields 4B and F14, respectively, representing five individual plants per field; (-): negative control (water); C: positive control (sample ALF1060); M1 and M2: DNA size markers. The red arrow indicates the position of the SRAV-specific, 843 bp band amplified with the ANPV_3 primers (Supplemental Table S3).

In an attempt to identify a possible arthropod vector for SRAV, two species of insects were collected in the same alfalfa fields (4B and 14B) where foliar samples were procured in August 2021; three pea aphids and 20 western flower thrips feeding on alfalfa were collected at the same time. All three aphids were bulked together into a composite sample, and the thrips were bulked into two composite samples according to the collection field: nine thrips from field 4B and eleven thrips from field 14B. Total nucleic acids were extracted from each of the composite samples according to the protocol described by Dellaporta et al. [24] and subjected to RT-PCR testing for the same seven viruses as all the foliar alfalfa samples. A prominent 843 nt SRAV-specific PCR band was amplified from the composite, eleven-thrip 14B sample (Figure 4) and, after Sanger sequencing, confirmed to represent the SRAV sequence 99.9% identical to the HTS-derived SRAV sequence (see Table 3). However, this SRAV-specific band in the 14B thrips sample appeared to be substantially weaker than the corresponding band in plant samples (Figure 4). Three additional virus-positive bands were amplified from both the 4B and 14B thrips samples, AMV-, BLRV, and MsAPV1-specific (not shown), suggesting ingestion of the virus-specific RNAs for these four viruses by the thrips and confirmed to represent the respective viruses following Sanger sequencing of the RT-PCR products (Table 3).

## 4. Discussion

The alfalfa virome from the samples collected in southern Idaho revealed, for the most part, a relatively common profile of viruses characteristic of *M. sativa* (L.), such as AMV and BLRV with positive-sense ssRNA genomes, and four persistent viruses with dsRNA genomes: MsAPV1, MsAPV2, MsDPV1, and MsAV1 (Table 2). In this respect, HTS data confirmed the presence of the common alfalfa viruses in the U.S., as described previously [2,4,19]. This virus profile is also consistent with the prevalence of AMV, BLRV, and persistent viruses in alfalfa crops in Europe, Australia, China, and South America [5,8,9,13,18]. However, one specific exception to this commonality was a new virus discovered in alfalfa crops grown along the Snake River valley in Idaho: SRAV. The 11,745 nt SRAV genome of encodes a single polyprotein of 3835 aa and displays a genome architecture consistent with flavi-like viruses (Figure 2). Phylogenetic analysis of the RdRP, the standard, universal phylogenetic marker for RNA viruses, placed SRAV within the large lineage of flavi-like viruses, making it the first member of this virus clade identified to infect plants. However, if SRAV is indeed a flavi-like virus, it is a highly divergent one. The SRAV polyprotein contains only two identifiable domains, namely RdRP and serine protease (Figure 2). Most unusually, SRAV lacks a detectable helicase, making it the first such case for an RNA virus with a genome larger than 6 kb. Helicases are readily identifiable not only by overall sequence conservation but also through diagnostic amino acid motifs, such as the P-loop (Walker A motif) [34]. The absence of such motifs in SRAV makes it highly unlikely that a divergent helicase was missed in our analysis. Conceivably, SRAV might recruit a cellular RNA helicase to facilitate virus genome replication. Another readily detectable domain encoded by flavi-like viruses is the methyltransferase domain of the capping enzyme [35]. No sequences similar to this domain were detected in the SRAV polyprotein either. Finally, although the overall genome organization of SRAV is compatible with the location of structural protein-coding sequences near the 5′-end of the genome, similar to the genome organization of flavi-like viruses, no similarity to any virus structural proteins were detected in the SRAV polyprotein. Together with the long branch formed by the SRAV RdRP in the phylogenetic tree (Figure 3), this lack of detectable homologs of most proteins of flavi-like viruses indicates a long, complex evolutionary history since the radiation of SRAV from the common ancestor with other flavi-like viruses. The substantial deviation of SRAV from the common genome layout of flavi-like viruses could conceivably be associated with the adaptation of this virus to reproduction in plants or, more likely, to the dual plant and insect host range. The ancestor of SRAV was potentially an insect virus that subsequently adapted to reproduce in plants. Identification of additional viruses related to SRAV should help predict the structures and functions of the large portions of the polyprotein that currently remain uncharacterized. In particular, such analysis should clarify whether SRAV encodes highly divergent versions of structural proteins of flavi-like viruses or, considering the plant host, unrelated structural proteins.

In the megataxonomy of viruses recently adopted by the International Committee on Taxonomy of Viruses (ICTV) [36], flavi-like viruses comprise the class *Flasuviricetes* within the phylum *Kitrinoviricota* of the kingdom *Orthornavirae*. Given the distant relationship between SRAV and other flavi-like viruses, this virus is likely to become the founder of a new family or even order within *Flasuviricetes*. However, taxonomic assignment will be possible only once additional viruses related to SRAV are discovered.

The host range of flavi-like viruses is currently almost exclusively confined to vertebrates and arthropods [37]. Some flaviviruses are known to cycle between vertebrate and arthropod (ticks and insects) hosts, with arthropods serving as virus vectors, whereas others exclusively infect arthropods [38,39]. One flavi-like virus, soybean cyst nematode virus 5 (SbCNV-5), was reported to infect a nematode [40], but so far, no flavi-like virus has been reported in plant hosts [37,38,39]. A dsRNA virus denoted as gentian Kobu-sho-associated virus (GKaV), with a monopartite genome of ca. 23 kb, encodes an RdRp that is most closely similar to RdRPs of pestiviruses [41,42]. Subsequently, it has been argued that GKaV might actually have an ssRNA genome and could be a flavi-like virus, possibly even within the genus *Pestivirus* [40]. However, both the genome structure and the evolutionary relationships of GKaV remain to be clarified. The possibility that SRAV is an arthropod virus that was accidentally picked up as a contamination during sampling and sequencing does not appear plausible, given the high abundance of the virus in multiple alfalfa samples—21 of the 41 symptomatic plant samples collected (Table 3). A far more likely scenario is an arthropod vector of SRAV, which is consistent with the observation of a weak, virus-specific RT-PCR band in a bulked sample of eleven thrips.

Of interest is an apparently wide prevalence of SRAV in alfalfa fields along the Snake River valley in the two counties of Idaho sampled over a period of two years, which are located on the opposite sides of the river, approximately 80 km apart (Table 1, Table 2 and Table 3). This wide spread of SRAV in south-central Idaho alfalfa crops seems to suggest that an efficient vector is present in this area and involved in SRAV epidemiology. With respect to prediction of possible vectors for SRAV, prevalence and dynamics of other vector-transmitted viruses found in the same alfalfa fields, such as AMV and BLRV, are likely to be informative. All samples from the two youngest alfalfa fields (4B and 14B) planted in 2020 were found to be negative for AMV in July 2021, but the proportion of AMV-positive samples increased in alfalfa stands of two years and older for fields 7C (planted in 2017) and 59A (planted in 2013) (see Table 3 and Supplementary Table S4). Similarly, samples from the two youngest alfalfa fields (4B and 14B, planted in 2020) had lower proportions of BLRV-positive samples than samples from fields 7C (planted in 2017) and 59A (planted in 2013) (Supplementary Table S4). This difference in virus prevalence in alfalfa fields planted in different years between aphid-transmitted AMV and BLRV, on the one hand, and SRAV, on the other hand, might indicate a different mode of transmission for SRAV that remains to be elucidated. Arthropod transmission is well-documented for flavi-like viruses infecting vertebrates. Analogously, arthropods are likely to serve as vectors for the plant-infecting SRAV, especially given the detection of SRAV sequences in thrips (Table 3, Figure 4, Supplementary Table S4).

The effects of viruses on the yield and quality of the alfalfa hay and on the overall economics of hay production is an understudied area [4,5]. All SRAV-positive samples analyzed in this work (Supplementary Table S4) presented as mixed infections with other common viruses, such as AMV and/or BLRV, not allowing for association of SRAV with specific virus-like symptoms in alfalfa. The possible causative role of SRAV in any symptom induction thus requires the fulfillment of Koch’s postulates for SRAV. The fact that SRAV was found in alfalfa crops does not suggest that alfalfa is the only host for this virus in the area, and a search for other agricultural and, perhaps, environmental hosts could be warranted. Initial attempts to mechanically transmit SRAV to *N. benthamiana* in a greenhouse experiment were unsuccessful but should be extended to other receptor hosts. In a preliminary experiment, SRAV was detected by RT-PCR in Western flower thrips collected in the same alfalfa fields where SRAV-positive plants were found (Figure 4). This RT-PCR amplification suggests that SRAV can be ingested by thrips during their feeding on alfalfa, along with other viruses. Although such ingestion does not, by itself, demonstrate transmission of these viruses by thrips, especially for the vertically transmitted MsAPV1, it seems suggestive in the case of SRAV, justifying further experiments on possible transmission of SRAV by thrips.

## 5. Conclusions

In this work, we reported the genome sequences and some biological characteristics of a highly unusual +RNA virus, SRAV, that commonly infects alfalfa in south-central Idaho. The overall organization of the 11,745 nt genome of SRAV and phylogenetic analysis of the RdRP suggest an evolutionary relationship between SRAV and flavi-like viruses. However, SRAV is only distantly related to these viruses and is, so far, unique among +RNA viruses with genomes larger than 6 kb apparently lacking a helicase domain. Thus, SRAV is likely to become the founding member of a new virus family or order once additional related viruses are discovered. To the best of our knowledge, SRAV is the first flavi-like virus detected in plants. SRAV sequences were also identified in thrips, suggesting that these insects might serve as vectors for this virus. The unique features of SRAV might have evolved as an adaptation to reproduction in plants or in both plants and insects.

## Figures and Tables

**Table 1:** A brief summary of the samples collected for alfalfa virus testing in Minidoka and Twin Falls counties, Idaho, in 2020–2021, with origins and type of sample, as well as the method used to detect viruses.

Location/Sample Type	Collected Specimens	Time of Collection	Number of Samples	Method of Analysis
Location 1: Minidoka (ID), leaf tissue	Alfalfa	July 2020	6	HTS, RT-PCR
Location 2: Twin Falls (ID), leaf tissue	Alfalfa	July 2021	25	RT-PCR
Location 2: Twin Falls (ID), leaf tissue	Alfalfa	August 2021	10	RT-PCR
Location 2: Twin Falls (ID), insects	Thrips	August 2021	2	RT-PCR
	Aphids	August 2021	1	RT-PCR

**Table 2:** Summary of the high-throughput sequencing (HTS) results and virus specificity assignments for the five alfalfa samples collected in July 2020, arbitrarily labeled as ALF1059 to ALF1071.

Viruses ^(a)^	ALF1059		ALF1060		ALF1061		ALF1067		ALF1071	
	Contig Size, nt ^(b)^	Mapped Reads, # ^(c)^	Contig Size, nt	Mapped Reads, #	Contig Size, nt	Mapped Reads, #	Contig Size, nt	Mapped Reads, #	Contig Size, nt	Mapped Reads, #
***AMV***										
RNA1	3620	*952,345*	3627	*559,215*	3629	*859,660*	3630	*680,142*	3541	1224,303
RNA2	1867	*361,173*	2497	*169,385*	2575	*466,668*	2563	*559,374*	2572	512,950
RNA3	2029	*507,736*	2012	*182,108*	2010	*732,928*	1999	*353,662*	2017	750,965
***BLRV***	-	*2*	-	*4*	-	*12*	4162	*5895*	5895	3207
***MsAPV1***										
RNA1	1903	*5378*	1996	*10,463*	1870	*11,193*	1774	*14,650*	1633	22,723
RNA2	1790	*422*	1679	*725*	1793	*551*	1785	*688*	1757	1001
***MsAPV2***										
RNA1	-	*0*	1902	*1236*	-	*2*	-	*0*	-	0
RNA2	-	*0*	1771	*161*	-	*0*	-	*0*	-	0
***MsDPV1***										
RNA1	-	*2*	-	*0*	-	*20*	-	*0*	1552	45
RNA2	496	*4*	-	*0*	1503	*57*	-	*0*	1353	134
***MsAV1***	2905	*154*	3369	*177*	3386	*293*	3415	*193*	-	0
***SRAV***	**-**	***0***	**11,803**	***1269***	**7788**	**275**	**-**	***0***	**11,797**	**1662**

^(a)^ AMV = alfalfa mosaic virus, BLRV = bean leafroll virus, MsAPV1 = Medicago sativa alphapartitivirus 1, MsAPV2 = Medicago sativa alphapartitivirus 2, MsDPV1 = Medicago sativa deltapartitivirus 1, MsAV1 = Medicago sativa amalgavirus 1, SRAV = ‘Snake River alfalfa virus’. ^(b)^ The largest virus-specific contig assembled for the sample. ^(c)^ The number of paired-end 300 bp individual reads mapped to the largest contig.

**Table 3:** Summary of virus-positive tests based on RT-PCR for all alfalfa samples collected in 2020 and 2021. The numerator designates the number of positives out of the total number of samples tested, i.e., the denominator. AMV = alfalfa mosaic virus, BLRV = bean leafroll virus, MsAPV1 = medicago sativa alphapartitivirus 1, MsAPV2 = medicago sativa alphapartitivirus 2, MsDPV1 = medicago sativa deltapartitivirus 1, MsAV1 = medicago sativa amalgavirus 1, SRAV = ‘Snake River alfalfa virus’.

Collection Date/Field	Planting Year	AMV-R2	BLRV	MsAPV1-R1	MsAPV2-R1	MsDPV-R1	MsAV1	SRAV
*July 2020, Rupert, ID*								
NE	2017	6/6	4/6	6/6	2/6	3/6	5/6	**4/6**
*July 2021, Kimberly, ID*								
4B	2020	0/5	2/5	5/5	2/5	3/5	2/5	**3/5**
14B	2020	0/5	0/5	3/5	1/5	4/5	3/5	**2/5**
23S	2019	1/5	3/5	5/5	1/5	1/5	2/5	**3/5**
7C	2017	5/5	4/5	5/5	2/5	1/5	2/5	**4/5**
59A	2013	3/5	5/5	5/5	1/5	0/5	1/5	**3/5**
*August 2021, Kimberly, ID*								
4B	2020	5/5	5/5	5/5	3/5	3/5	3/5	**1/5**
14B	2020	3/5	5/5	5/5	2/5	1/5	4/5	**2/5**

**Table 4:** Summary of an experiment testing mechanical transmissibility of Snake River alfalfa virus (SRAV) and alfalfa mosaic virus (AMV) when inoculated into *Nicotiana benthamiana* plants under greenhouse conditions. *N. benthamiana* upper, non-inoculated leaves were sampled at 5 and 8 weeks post-inoculation (wpi) and tested using RT-PCR with primers listed in Supplemental Table S1. The numerator designates the number of positive samples of the total number tested, i.e., the denominator. AMV = alfalfa mosaic virus, SRAV = ‘Snake River alfalfa virus’.

Sample Origin (Field and ID)	5 wpi		8 wpi	
	AMV	SRAV	AMV	SRAV
4B-1	**3/3**	0/3	**3/3**	0/3
4B-2	**2/3**	0/3	**3/3**	0/3
4B-3	0/3	0/3	0/3	0/3
4B-4	**3/3**	0/3	**3/3**	0/3
4B-5	**3/3**	0/3	**3/3**	0/3
14B-1	**3/3**	0/3	**3/3**	0/3
14B-2	0/3	0/3	**3/3**	0/3
14B-3	0/3	0/3	**3/3**	0/3
14B-4	0/3	0/3	**3/3**	0/3
14B-5	**3/3**	0/3	**3/3**	0/3

## Data Availability

All data are available upon reasonable request.

## Supplementary Materials

The following supporting information can be downloaded at: https://www.mdpi.com/article/10.3390/v14061320/s1, Figure S1: Alignment of the RdRP-containing regions of the polyproteins for a subset of flavi-like viruses and SRAV, Figure S2: Secondary structure prediction for the portion of the SRAV polyprotein encoding the serine protease, Figure S3: Phylogenetic analysis of the RdRP domains of flavi-like like viruses and phylogenetic placement of the newly described Snake River alfalfa virus (SRAV); Table S1: Summary of primers used in this study and their sequences, Table S2: Summary of basic statistics of high-throughput sequencing data processing for each alfalfa sample, Table S3: List of the viruses and corresponding genome components identified in the 2020 alfalfa samples by high-throughput sequencing, Table S4: RT-PCR-based testing of individual alfalfa samples collected in 2020 and in 2021.
