# Supplementary material for: A Novel Flavi-like Virus in Alfalfa (Medicago sativa L.) Crops along the Snake River Valley
Source: Viruses. 2022 Jun 16;14(6):1320. doi: 10.3390/v14061320 (PMC9228291; doi:10.3390/v14061320)
Supplement: Supplementary file 1 [file viruses-14-01320-s001.zip › viruses-1751740-supplementary/KarasevFinal_zip/Supp Figure S2.pdf]

```
>PF05579.16 ; Peptidase_S32 ; Equine arteritis virus serine endopeptidase S32
Probab=93.86  E-value=1.5  Score=36.69  Aligned_cols=105  Identities=19%  Similarity=0.149  Sum_probs=0.0  Template_Neff=9.700

Q ss_pred              ceeEEEEEccCCcceEEEEhhHHHHhccccccccccccceeeeeccccchhcchh-------ccccccccccCCceeEEE
Q SRAV           1791 RGKLSVLTEQGEGSQIMVTAAHAWRYGIDSFTGNLRDYPTVISAASNRSSLFRED-------MVSEGEFDYTNGRQCVVA  1863 (3835)
Q Consensus      1791 rgklsvlteqgegsqimvtaahawrygidsftgnlrdyptvisaasnrsslfred-------mvsegefdytngrqcvva  1863 (3835)
                      .|.=.++...|.+  .++|++|... +-.-.-..-.....+.....+.-.+++-+       ...-+ -....|..+.+.
T Consensus       113 ~gsG~~i~~~g~~--~ilT~~Hvv~-~~~~~v~~~~~~~~~~~~~~~DlAll~~~~~~~~~~~~~l~-~~~~~g~~v~~~  188  (296)
T RPOA_LDVC/1418  113 SGSGGVFTINGNP--VVVTASHLLS-DGKARVSCVGFSQCLDFKCAGDYAFARVANWKGDAPKAELS-HRRGRAYCSPLV  188  (296)
T ss_pred              cEEEEEEEeCCCE--EEEEchhhcc-CCCEEEEeCCceEEEEecCCCCEEEEEEcCCcCCCCccccc-cCCCccEEEEEE


Q ss_pred              EeEEEecCCCCceEEEEEEEeeecCccCCccEEcCCcceeeee
Q SRAV           1864 RNVYKTDSDRYMVVSVDVMNYGGYSGGPVVTSDGEFLGIC  1903 (3835)
Q Consensus      1864 rnvyktdsdrymvvsvdvmnyggysggpvvtsdgeflgic  1903 (3835)
                      ........+.|.....+    .|.||||++..+|+++||.
T Consensus       189 g~~~~~~~~~~~~~~~~~~----~G~SGgpv~~~~G~viGi~  224  (296)
T RPOA_LDVC/1418  189 GLSLDLLGKNSAFCFTK----CGDSGSPVVDEDGNLLGIH  224  (296)
T ss_pred              EecccccCCCeeEeeeC----CCCCCcEEECCCCCEEEEE
```
