# Supplementary material for: A Novel Flavi-like Virus in Alfalfa (Medicago sativa L.) Crops along the Snake River Valley
Source: Viruses. 2022 Jun 16;14(6):1320. doi: 10.3390/v14061320 (PMC9228291; doi:10.3390/v14061320)
Supplement: Supplementary file 1 [file viruses-14-01320-s001.zip › viruses-1751740-supplementary/KarasevFinal_zip/Supplemental Table S4 (revised).pdf]

Supplemental Table S4. RT-PCR based testing of individual alfalfa samples collected in 2020 and in 2021. For multicomponent virus genomes, only one genome component was tested for as indicated. Numerator, "+" indicates virus-positive status determined by RT-PCR, "-" indicates virus-negative status; denominator, "+" indicates the PCR product was sequenced and found 98.5-100% identical to the virus sequence. Samples are divided in three groups by the date of collection.

Viruses	AMV-R2	BLRV	MsAPV1-R1	MsAPV2-R1	MsDPV1-R1	MsAV1	SRAV
July 2020 samples							
ALF1059	+/+	+/+	+/+	-	+/+	+/+	-
ALF1060	+/+	-	+/+	+/+	-	+/+	+/+
ALF1061	+/+	+/+	+/+	-	+/+	+/+	+/+
ALF1063	+/+	-	+/+	+/+	-	+/+	+/+
ALF1067	+/+	+/+	+/+	-	-	+/+	-
ALF1071	+/+	+/+	+/+	-	+/+	-	+/+
July 2021 samples							
4B#1	-	+/+	+/+	+/+	-	-	-
4B#2	-	-	+/+	+/+	+/+	+/+	-
4B#3	-	+/+	+/+	-	-	-	+/+
4B#4	-	-	+/+	-	+/+	+/+	+/+
4B#5	-	-	+/+	-	+/+	-	+/+
7C#1	+/+	+/+	+/+	+/+	-	-	+/+
7C#2	+/+	+/+	+/+	-	-	-	+/+
7C#3	+/+	+/+	+/+	-	-	+/+	+/+
7C#4	+/+	+/+	+/+	+/+	-	-	+/+
7C#5	+/+	-	+/+	-	+/+	+/+	-
14B#1	-	-	-	-	-	-	-
14B#2	-	-	+/+	-	+/+	+/+	-
14B#3	-	-	-	-	+/+	+/+	-
14B#4	-	-	+/+	-	+/+	+/+	+/+
14B#5	-	-	+/+	+/+	+/+	-	+/+
23S#1	+/+	-	+/+	+/+	-	-	-
23S#2	-	+/+	+/+	-	-	+/+	+/+

23S#3	-	-	+/+	-	-	-	+/+
23S#4	-	+/+	+/+	-	+/+	-	+/+
23S#5	-	+/+	+/+	-	-	+/+	-
59A#1	-	+/+	+/+	-	-	-	+/+
59A#2	+/+	+/+	+/+	-	-	+/+	+/+
59A#3	+/+	+/+	+/+	+/+	-	-	-
59A#4	+/+	+/+	+/+	-	-	-	-
59A#5	-	+/+	+/+	-	-	-	+/+
August 2021 samples							
Aphids4B	-	+/+	-	-	-	-	-
Thrips4B	+/+	+/+	+/+	-	-	-	-
Thrips14B	+/+	+/+	+/+	-	-	-	+/+
4B-1	+/+	+/+	+/+	+/+	+/+	-	-
4B-2	+/+	+/+	+/+	+/+	+/+	+/+	-
4B-3	+/+	+/+	+/+	-	+/+	+/+	-
4B-4	+/+	+/+	+/+	-	-	-	+/+
4B-5	-	+/+	+/+	+/+	-	+/+	-
14B-1	+/+	+/+	+/+	-	+/+	-	-
14B-2	-	+/+	+/+	-	-	-	+/+
14B-3	-	+/+	+/+	+/+	-	-	-
14B-4	-	+/+	+/+	+/+	-	-	+/+
14B-5	+/+	+/+	+/+	-	-	-	-
