# Supplementary figures and images for: A Novel Flavi-like Virus in Alfalfa (Medicago sativa L.) Crops along the Snake River Valley
Source: Viruses. 2022 Jun 16;14(6):1320. doi: 10.3390/v14061320 (PMC9228291; doi:10.3390/v14061320)

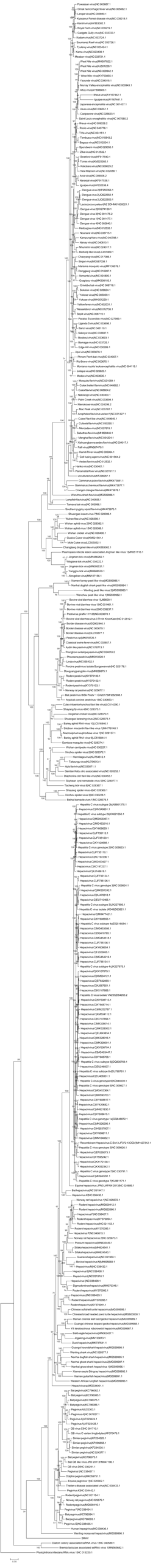

Supplement: Supplementary file 1 [file viruses-14-01320-s001.zip › viruses-1751740-supplementary/KarasevFinal_zip/Supp Figure S3.pdf]
